# Annotations

**Published:** 1902-01-04

**Authors:** 


### Annotations.

### Alcohol and the Race.

Few questions illustrate more markedly how widely people may differ, even with the same facts before them, than the perennial problem of the influence of alcohol on the race. On the one hand, we have the events which we are told are occurring in France, and much that we see going on before our eyes in this country, the sickly infants, the puny children, the stunted men and women, the feeble intellects, the morbid nervous systems which occur among the offspring of drinking parents, in addition to the comparative sterility of the drunkards themselves, all fixing upon our minds a miserable picture of the degradation and ultimate extinction of an alcohol-drinking race. On the other hand, we have the facts put forward by those who hold that alcohol like every other evil influence to which a race may be exposed tends by weeding out the unfit to produce in the end a hardy breed, a people immune to the evils to which so many of their predecessors have succumbed. Thus, while one party holds that the free use of alcohol is destructive to a nation, the other teaches that only by its use and the consequent weeding out of the susceptible can a nation rise superior to its ever present attraction. In a recent article in the *Archives Générales de Médecine* Dr. Lucien Mayel has drawn a striking picture of the depopulation and the degradation of physique which are going on in certain parts of France under the influence of alcohol. Nor need we go to France for evidence to this effect. In the high mortality attending certain trades in which drinking habits prevail we have evidence enough of the dangers of alcohol to the individual, while a very moderate acquaintance with town life will convince the most sceptical of the truth of Dr. Mayel's description of its effect upon a nation. The fact is the difference between different observers to which we have alluded arises from difference in point of view. No one pretends to deny the deteriorating influence of alcohol on those who prove susceptible to it. But Dr. Archdall Reid and those with him regard the matter as a question of heredity and evolution. To them the problem is one that must work itself out during many generations, and if our main responsibility is for those who come after us, they are doubtless in the right. Most of us, however, feel pretty strongly that our responsibility for the woes of our own generation is quite as much as we can bear, and that even at the risk of our great-great-grandchildren it is worth while to do our best to check the prevailing intemperance of the present day.

### The Prospects of the Midwives Bill.

There can be no doubt that the practitioners who voted for the successful candidates at the recent election to the General Medical Council intended their votes as a protest against all legislation which might have the effect of giving any legal position to the midwives. It seems probable, however, that in thus supporting an impossible programme these gentlemen will have actually facilitated the passage of the very Bill to which they are so opposed. There is every reason to believe that the Bill of the Midwives Committee will be brought forward again in the coming session; that it will receive such an amount and such a kind of support that Parliament will feel that something must be done; and that, unless some definite and well-thought-out scheme be brought forward in opposition to it, that "something" will turn out to be the Bill pretty much as it stands. The only other definite and well-thought-out scheme which could pretend to any authority was that of the British Medical Association, and, although we do not think it ever had or would have the slightest chance of passing, it had its good points, and if it could even now be presented as expressing with any approach to unanimity the feeling of the medical profession, it would at least provide a bundle of alternative clauses to be put forward as opportunity arose. All that, however, is knocked on the head. The medical profession has by its vote decided that it will have nothing to do with legalising the practice of midwifery by any but fully qualified practitioners of medicine. The Association then could not bring forward its Bill with the slightest pretence of speaking in the name of the profession, and almost certainly will not bring it forward at all. The most effectual form of obstruction, namely an alternative scheme, to be discussed clause by clause in committee, will be removed; the question before the House will probably be put "Aye" or "No," and if so we shall be greatly surprised if the Bill of the Midwives Committee does not walk through. For that result the medical profession will have themselves to thank, in that they have placed themselves, on this question, in irreconcilable opposition to the inevitable.

### Pearls.

However useful pearls may be in the treatment of various feminine neuroses and in soothing those angry passions which lead to "nerve storms" of various descriptions, it is not because of their therapeutic efficacy that we refer to them at this moment so much as to draw attention to certain researches which have recently been made by a French naturalist, M. Raphael Dubois, in regard to their origin. It appears, according to his investigations, that the pearl-bearing mollusks are liable to be infested with minute distomata, marine worms which, during a particular stage of their growth, become encysted in a complete calcareous envelope, which is in fact a young pearl. At this stage the worm can be discovered by decalcifying the pearl with dilute hydrochloric acid. The young pearl is, in fact, a protection to the worm much as the cocoon is to the silkworm, and there is this further analogy between them that as the silkworm moth destroys the cocoon in making its escape, so when the time arrives for the pearl-worm, if one may so call it, to move its quarters, the pearl loses its polish, decays, and falls to pieces, while the worm escapes and fulfils its destiny, whatever that may be. But supposing the worm should die, then quite another series of events takes place. The little pearl does not dissolve. On the contrary, the calcareous deposit continues to grow, and if the mollusk should live long enough, layer after layer is laid on until we get a real pearl, often a pearl of great price, as we discover to our cost when we pay a visit to the jeweller's. Still, after all, this beautiful jewel is but the coffin of a worm. Its success as a pearl comes from its failure as a parasite.

